# Diphtheria

**Published:** 1899-12-30

**Authors:** 


### DIPHTHERIA.

In the last of the Harben Lectures Professor W. R. Smith, after pointing out the great diminution in the fatality of the disease since the introduction of the antitoxin treatment, went on to show that, although there was but little probability of a definite case of diphtheria now remaining unnotified for any length of time and thus becoming a source of danger to the community, such a danger constantly arose in connection with mild and unrecognised cases; as, for example, when one member of a family having contracted diphtheria infected other members or other people before definite symptoms necessitating isolation arose. The same might occur among school-children, the infection being distributed before the disease became obvious in the first case. Convalescents also were often a source of danger, for, although clinically cured, they might still retain diphtheria bacilli on their fauces, and might communicate the disease to others on their return from hospital. Various measures were suggested to obviate the evils so caused, but he said that the measure which above all he desired to urge was the prophylactic use of antitoxin. It had been shown that by the use of a moderate dose it was possible to secure immunity for at least a month, and that, even after that period, the disease if acquired ran a mild course. This prophylactic use of the serum had, he said, been largely taken advantage of on the Continent and in America, though comparatively little attention had been given to the subject in this country. A solution of the antitoxin could, he said, be prepared of such a strength that the prophylactic dose would be only one-fifth of a cubic centimetre, and he felt certain that by the adoption of this means the amount of diphtheria might be materially diminished.

